# ICT Learning by Older Adults and Their Attitudes toward Computer Use

**DOI:** 10.1155/2015/849308

**Published:** 2015-08-05

**Authors:** Antonio González, María Paz Ramírez, Vicente Viadel

**Affiliations:** ^1^Department of Social Services, Provincial Council of Cuenca, C/Sargal s/n, 16001 Cuenca, Spain; ^2^Cáritas Diocesana de Cuenca, Avenida República Argentina No. 27, 16002 Cuenca, Spain

## Abstract

Information and communication technologies have proven to be an effective way of helping older adults improve independence outcomes, but such technologies are yet not widely used by this segment of the population. This paper aims to study computer use and senior citizens' attitudes toward computer technology in the context of a 20-hour course in basic skills. A questionnaire was used to conduct pre- and postcourse analyses with a sample of 191 adults over the age of 60. The findings show that direct contact with computers generates more positive attitudes toward computer use and also positive relationships with attitudes, user behavior, training expectations, and self-confidence. Results are discussed in the light of need-to-know attitudes toward computer use and training in new technologies as an opportunity for life-long learning and for improving quality of life in old age.

## 1. Introduction

The study of computer use by older people is currently booming, as this group has significantly increased its use of information and communication technologies (ICT) in daily life, on both personal and professional levels [1]. In this respect, the advantages of using new technologies to develop social relationships, leisure and entertainment opportunities, and life-long learning habits and access services and care could be considered factors for successful autonomy and aging in the life of older people [2].

“As the availability of electronic resources for older adults increases, the quality of the aging experience will be enhanced” [3, page 34] and in order to achieve involvement and counteract resistance to using these technologies, we need to bridge the “digital gap” experienced by older users by providing interfaces and tools adapted to suit their physical and cognitive characteristics [4–6].

Research is inconclusive as to the positive psychosocial effects of ICT use on senior well-being [7, 8] and as a result, its influence on the improvement of cognitive skills such as memory and attention is considered to be very weak [9]. However, contrary to popular belief, older people respond positively to using computers, leading to favorable changes in their interests and confidence due to the recognition of these technologies as beneficial tools and as a result of using them in training and learning programs [10–12]. Along these same lines, the results of specific studies note that cognitive abilities alone are not enough to predict older adults' computer use but that attitude variables like computer self-efficacy and computer anxiety must also be considered [13, 14].

As a result, attitudes and needs must be considered when designing adequate ICT teaching programs, taking into consideration aspects relating to perceived benefits and difficulties [15] and usefulness and barriers to use [16], as well as confidence and ease when learning [17]. Specifically, given that seniors require more time to acquire knowledge, make more mistakes, and need greater support, teaching methods must target these challenges and instructions must be task-orientated, involving older learners by using highly interactive teaching methods [18, 19]. Suitable training and support systems are required according to seniors' objectives, abilities, and experience, so as to reduce anxiety about using computers and to promote motivation, especially in the initial stages of learning [20, 21].

Therefore, the specific aim of this study is to explore the behavior and attitudes toward computer technology among older people in a course for learning basic computer skills, which was structured and specifically designed for their level of education and knowledge.

Now that ICT access has increased significantly among all segments of the population, there is a need to research its learning and use among seniors, in order to allow us to understand their needs as a heterogeneous social group with diverse interests, education, health, and socioeconomic levels. Greater attention must be paid to how learning environments can affect the processes of using these technologies. Using this need as a starting point, this study aims to understand computer use among seniors.

## 2. Method

### 2.1. Participants

The sample for this study was obtained through the “Cuenc@enRed” network of computers and an Internet course that took place from February to May 2011. The sample included enrolled elders in this activity carried out by the Social Services of the Provincial Council of Cuenca (Spain) and the “Insula Barataria” Foundation of the Autonomous Community of Castilla-La Mancha (Spain). The course was offered for a total of 25 senior citizen centers within the context of a formative program about active ageing that included contents adjusted for elders with limited or nonexistent ICT skills

This program was designed as a 20-hour course to learn basic computer skills, with 30 subsequent hours of tutorials to consolidate these skills, for a total of 50 hours. The program was specifically structured to be suitable for teaching older people and to address the basic knowledge of computers, files, the Internet, e-mail, chatting, and word processing. At the beginning of the training program, participants were given simple manuals designed to provide course contents, including step-by-step instructions and numerous illustrations. The manual was made up of basic information to understand parts of the hardware (such as the keyboard or the mouse), functions of the operating system (Windows), Internet, e-mail, and word processor. It was designed to teach ICT skills to elders [22]. The course consisted of a total of 20 hours, imparted as 2-hour lessons each day for the first two weeks. The rest of the training, which was focused on consolidating the knowledge acquired in the first two weeks, was taught in 2-hour tutorials, two days a week, until reaching a total of 30 hours.

In order to understand the attitudes toward computers and to analyse whether direct contact with the computer generates more positive attitudes, a total of 191 seniors who participated in the first session of the course answered a questionnaire before taking the course. The same questionnaire was completed by the 173 seniors who participated in the last session, after completing the 20-hour course. 18 participants did not finish the course; they dropped out during the training period. Family and health related issues were the reasons for their dropout.  Table 1 shows the distribution of the subjects in the sample, according to the background information that was considered: place of residence, gender, age, studies completed, level of income, marital status, and self-confidence. Ages ranged from 60 to 89 years; the mean was 68.65 and the standard deviation was 6.29. Under place of residence, the percentages were distributed according to whether the sample came from senior centers in towns with over 2500 inhabitants or centers in smaller locations, leaving us with two groups of subjects. It is worth highlighting that 60% of the samples were 60–70 years old, suggesting that this age group is the most willing to participate in this kind of activity, being the group that is most active and most interested in new technologies. Almost 80% of the sample had received elementary education and nobody in this sample declared a high level of income. Similarly, more than 85% of the elders considered themselves self-confident individuals. Moreover, the sample was asked about the economic level they believed they had. The answers could only be one of the following options: high, average, low, or very low.

### 2.2. Procedure and Measures

The class instructors responsible for teaching during the course had a higher academic level in different university disciplines and they took part in a 4-hour training programme to learn how to teach ICT to old people; specifically, they learnt how to teach ICT skills taking into account the cognitive abilities of elders [19]. The instructors were also given specific and precise instructions so they knew how to distribute the surveys. They were responsible for handing out the questionnaires and giving instructions for its correct completion, and they asked the participants to complete it anonymously and on their own.

To carry out this study, a questionnaire designed to measure different aspects related to the study was prepared. Specifically, the following questions were included in the survey.

*Background Information and Computer/Internet Use Variables*. The following variables were included: place of residence, gender, age, level of education, marital status, and general level of income declared. Questions were also included concerning the frequency of computer use and Internet access, perception of the difficulty of the course, and whether or not they believed themselves to be self-confident.

*Senior Citizens' Attitudes toward Computers (SAC)*. To measure seniors' attitudes toward computers and toward learning basic computer skills, a specific scale was designed based on instruments used and validated in previous research on this subject (see the Appendix) [23–25]. With the aim of measuring these attitudes, various items used by these instruments were considered and adapted to the population participating in the study, which generally had a low level of education and little or no prior computer contact. To this end, a total of 26 questions were drafted addressing various attitudes concerning the effectiveness, ease, control, interest, and usefulness of computer use. Participants were evaluated on this scale at the start of the course and the evaluation was then repeated after the first 20 hours of teaching, together with an assessment of their satisfaction with the teaching received during those first two weeks of training. To make it easier for older people with low levels of education to answer the questions, they were kept simple, clear, and short using the four-point scale format (a lot, quite a lot, not much, and not at all).

## 3. Results

### 3.1. Behavioral Patterns of Computer Use by Older People

To find out how often older people use computers and their potential Internet access, two questions were posed about these issues: “How much time do you usually spend on the computer?” and “Do you normally use the Internet?” Results showed that 46.1% of the sample never uses a computer, 39.6% rarely, 12.4% sometimes, and 1.8% a lot. In this respect, the senior citizens participating in this study had hardly had any contact with computers, making this course their first opportunity for learning about and using a computer in most cases.

Regarding Internet access, 54.8% of the senior citizens said that they never use the Internet, 22.4% occasionally, 11.4% almost never, and 11.4% almost every day. Given these percentages, we can infer that some older people use the computer without ever accessing the Internet, as the percentage declaring that they have never accessed the net is significantly greater than the percentage declaring that they have never used a computer. Taken as a whole, the results for both questions reveal that the older people registered for this course have hardly had any prior contact with computers and the Internet and that, in most cases, this course represents their first opportunity for learning about and using these computer skills. On the other hand, the question “how do you think this course will be?” was introduced in the questionnaire to know what was the elders expectancy about the difficulty of the course. Results regarding the anticipated degree of difficulty of the course showed that 61.2% of the sample believed that the course would not be very difficult and 29% answered that it would be difficult.

Moreover, as it has been mentioned in the method section, we introduced a question regarding the self-confidence of every individual: “Overall, I consider myself a self-confident person.” As it is shown in Table 1, the vast majority of the elders thought they were self-confident individuals. On the other hand, as it is shown in Table 2, this variable had a positive correlation with favourable attitudes towards the use of computers.

### 3.2. Senior Citizens' Attitudes toward Computers

To evaluate the psychometric characteristics of the scale used to measure old people's attitudes towards computers, an exploratory factor analysis with varimax rotation was performed. After considering the reliability coefficients and the factor loadings of the 26 items that made up the scale, the statement “I think using the computers is very easy” was eliminated because its correlation coefficient was very low. The remaining 25 items were considered as a single factor scale. The final scores were constructed on the basis of simple summation across the different scores obtained on each item. A four-point scale was given to the answers (a lot = 4, quite a lot = 3, not much = 2, and not at all = 1), so the maximum score you can get on the scale is 100 and the minimum is 25.

Once the scores were analyzed, we found that 71 was the highest score and 31 was the lowest. The mean score was 52.75 and the standard deviation was 7.07.

As to reliability, Cronbach's *α* coefficient was 0.68. The scale produced a positive correlation between computer use (*r* = 0.22) and Internet access (*r* = 0.25), demonstrating that the set of 25 items had a certain degree of concurrent validity.

Table 2 shows the Pearson correlation matrix between the different variables taken into consideration in this study. The values of the correlations can be deemed moderate and, even in some cases, moderate-to-high and almost all of the variables are interrelated.

When observing the results obtained, it is worth highlighting the significant correlations between the SAC scale and other variables. Favourable attitudes toward computers correlate to computer use, frequency of Internet access, and self-confidence. Expectations of course difficulty correlate negatively to attitudes, computer use, and frequency of Internet access, meaning that the older people who viewed computers least favourably and who used it least believed that the course would be most difficult. Finally, those with the most self-confidence demonstrated a more favourable attitude toward computers, as shown by the positive correlation found between the SAC scale and their declaration of self-confidence.

One of this study's fundamental objectives was to examine whether seniors' attitudes toward computers were influenced by their contact with the computer during the 20-hour course. To answer this question, the 173 seniors who participated in the last session, after the first two weeks of training when the basic course was ending, were reassessed in all of the variables mentioned. The data obtained was used to perform an analysis *t*-test for related samples, resulting in significant differences in the scores obtained on the scale at the start of the course and after it finished (*t* = −8.85; *p* < 0.0001). Comparing the mean sample scores on the attitudes scale at the start of the course (*M* = 52.74; SD = 7.07) and after it ended (*M* = 59.51; SD = 6.42), it can be said that attitudes seem to change favorably. This result would point to the SAC scale as a valid instrument for measuring attitudes of seniors toward computer use.

On the other hand, a series of *t-*test and ANOVA were carried out, with gender, place of residence, medium-low income, and age taken as independent variables and the scale of attitudes toward computers, questions concerning computer and Internet use, belief in the difficulty of the course, and self-confidence as dependent variables. These analyses only produced significant results for gender and place of residence affecting self-confidence. Specifically, men were more self-confident than women (*t* = 2.39; *p* < 0.05) and senior citizens from small towns of less than 2500 inhabitants were also significantly less sure of themselves than those from larger towns (*t* = 2.03; *p* < 0.05).

### 3.3. Evaluation of Learning about and Using Computers by Older People

To evaluate satisfaction with the basic 20-hour course received and to understand the reasons for the interest generated, as well as any learning difficulties, a total of 173 older people answered questions about their level of satisfaction and about the structure and usefulness of the course received. The results obtained reveal that 60.1% of participants expressed that the course duration was inadequate, while a total of 91.2% said that the schedule had been very convenient or convenient.

Concerning the difficulty of the subjects addressed, 39.4% declared that it had been difficult or very difficult while 60.6% thought it was not or not at all difficult. In terms of learning, 50.5% said that they had learned a considerable or large amount and 49.5% said that they had learned little. Finally, regarding usefulness 93.5% believed the knowledge gained would be useful or very useful and 73.5% believed they would continue to use what they had learned after the course.

During the last session, after the 50-hour training program, that is, after the 20-hour course and the 30 hours of tutorials which followed, a group interview was held at all of the centers, to find out what participants thought about their interest and involvement in the activities they had participated in. A trained researcher visited each center and carried out an interview in the form of a group discussion, posing four questions for the participants to discuss. This researcher was there to act exclusively as a moderator and to make note of senior citizens' answers to each of the questions. These questions were as follows: “Why are you interested in learning to use a computer? What do you need to learn in order to use a computer? What are the problems/difficulties/obstacles when learning to use a computer?” and “Ideas/suggestions to improve this course and make it more useful.” A total of 140 senior citizens participated in these discussions, 76 men and 64 women.

The analysis of the interviews data collection was carried out following the Grounded Theory procedure [26, 27]. Relevant concepts were identified and classified per classes of meaning derived from answers given to the set of questions. Open coding was conducted to categorize the interviews data and a coding system was developed. Data was then coded into one or more analytic categories by breaking them and constantly comparing new data with already coded data. Categories with similar properties were grouped and regrouped until no new categories were located. The resulting categories are shown in Table 3.

As it is shown in Table 3, the results obtained show that the participants considered the learning, the communication, the entertainment, and the need to be up to date the main reasons to learn how to use the computer. Based on the contributions of the older people who participated in the interviews, we can verify that learning how to use the computer and the difficulties it involves were an issue that manifested itself as significant in this context. Some of their contributions are identified as highlighting memory and mental agility, initiative or practice, and dedication. Moreover, participants felt that the difficulty using the mouse and keyboard or fear of breaking the computer is aspects related with the learning of how to use the computer. However, the elders believed that with the appropriate guidance and practice they can learn the skills that they need to use a computer.

## 4. Discussion

The present paper represents an exploratory study regarding Aging and New Technologies, from an empirical and applied perspective. The principle objective was to better understand old people's attitudes and behavioural patterns concerning learning and using computers through a basic course given at senior centers.

Firstly, this study found no significant correlations between sociodemographic variables and computer use. Although some studies suggest that age, education, and income are factors in an older person's interest in learning and using ICT [28, 29], the results are not conclusive in this respect, perhaps due to the influence of mediating variables, such as experience or cognitive skills [30] that characterize the significant variation between individuals in this population group.

The results obtained demonstrate that the attitudes expressed by seniors toward computers measured using the SAC scale correlate positively to computer use, frequency of Internet access, and self-confidence while the expectations of course difficulty correlate negatively to computer use and frequency of Internet access. We also found that senior citizens' attitudes toward computers changed in a positive manner following initial contact through their participation in the basic 20-hour course. Therefore, as with other studies relating to this subject, the results point to the fact that greater involvement and contact with computers lead to more positive attitudes toward using and learning to use them [20, 31]. In the same way, some studies conclude that older people involved in learning about computers become more confident and have greater levels of self-esteem [17, 32, 33].

The results of the group interviews data collection carried out at the end of the training program are consistent with previous studies about the reasons given by seniors regarding their participation in the program and their learning difficulties, in which older people said that learning new technologies and adapting to new times were their fundamental motives for taking part in this type of training, while memory and attention were the basic requirements for learning about new technologies [34, 35]. The results also coincide with the affective or emotional dimension involved in the regular use of technology, so “the adjustments must be based on a conception of technology which, in accordance to the principles of gerontology, should meet the wishes and needs of the people who will use it. Thus, technology should be accompanied by adequate training and ongoing support to use the devices effectively” [36, page 240].

These results have significant implications for the educational practice of senior citizens and for the organization of computer-training activities. They prove older people are not reluctant to learn about computers due to a belief that the learning process is complicated or inaccessible. On the contrary, they believe that they can learn so long as they remain healthy and have adequate levels of cognitive function. Accordingly, professionals who work directly with senior citizens must keep in mind that learning to use computers and using computers to organize various activities for older people can stimulate self-confidence and augment self-esteem and are therefore a tool that can be called upon in the promotion of health and active aging. In this respect, in the field of aging, professionals must keep in mind the implications of computer use for continuing education and for the general well-being of those populations of older people who have had limited access to educational training and resources.

This study is also an important contribution to the field of attitudes toward the use of computers and ICT learning in senior citizens, showing as it does that these attitudes are positively affected by basic training lasting 20 hours. This proves that attitudes are immediately modified after the first brief moment of contact with computers in populations of older people and not just through continuous usage. As such, this study contributes in a relevant way to the field of computer use by senior citizens, since it concludes that even older people with a low level of education and limited computer use modify their attitudes positively toward new technologies as a learning tool, as a way of relating to others, as entertainment, and as a way of keeping current. In this way, the structure of the formative program introduced in this paper can be considered suitable for the basic formation and the change of attitudes toward ICT of elders with a low social and educative level and scarce informatics skills. However, the methodological limitations in this study suggest that we should continue evaluating the suitability of the formative program with new studies and different samples of elders before it can be considered fully ready for its generalization and diffusion.

Finally, the findings of this study should be considered under certain methodological limitations. This study has been carried out using a questionnaire that was expressly designed to measure certain aspects of seniors' attitudes toward computers, during a training course and with a relatively homogeneous group of older people. Further research with diverse samples from this population segment must be carried out to contrast the reliability and validity of the scale used in order to improve it and to design an instrument for measuring the attitudes as to the various aspects of computer use. On the other hand, the reported differences between pre- and postmeasures using a single-group pretest-posttest design cannot unequivocally be attributed to the computer training due to the limits of the single-group design. Therefore, in further studies it might be a good measure to randomize the control group design so conclusions withdrawn are more accurate, carrying out statistics analysis that evaluate the covariance between variables and the influence of the mediator variables like experience or previous ICT skill might also be a good measure for a more accurate result.

This objective could lead to relevant conclusions about senior interests, preferences, intentions, and behaviour regarding ICT and its subsequent application for activity professionals in designing teaching programs for learning about using these kinds of technologies by older adults.

## Figures and Tables

**Table 1:** Distribution of the sample of older adults (*N* = 191).

Variable	Groups	Percentage
Place of residence	+ towns	54.8
	− towns	45.0

Gender	Men	53.9
	Women	45.7

Age	60–64	23.8
	65–69	38.8
	70–74	21.0
	75–79	9.3
	80+	7.0

Level of education	Read and write	12.6
	Elementary	76.2
	Middle-high	7.8
	Postsecondary	2.9

Level of income declared	High	0.0
	Average	44.7
	Low	45.7
	Very low	9.6

Marriage status	Married	70.8
	Widow	16.9
	Separated	5.5

Self-confidence	A lot	20.5
	Quite a lot	65.1
	Not much	13.5
	Not at all	0.9

**Table 2:** Correlations between the different variables (*N* = 191).

	(1)	(2)	(3)	(4)	(5)
(1) Computer use	—				
(2) Internet use	0.69^*∗∗*^	—			
(3) Expectation of course difficulty	−0.30^*∗∗*^	−0.24^*∗∗*^	—		
(4) Self-confidence	−0.07	0.07	0.04	—	
(5) Seniors' attitudes (SAC)	0.22^*∗∗*^	0.25^*∗∗*^	−0.32^*∗∗*^	0.22^*∗∗*^	—

^*∗∗*^ *p* < 0.01.

**Table 3:** List of categories according to the analysis (*N* = 140).

Questions	Categories
Why are you interested in learning to use a computer?	To learn new things To relate to and communicate with people Entertainment and leisure To be up to date

What do you need in order to learn to use a computer?	Own computer Practice and dedication Interest and initiative Memory and concentration

Difficulties encountered when learning to use a computer	Mouse and keyboard Illiteracy Memory and mental agility Fear of the computer or fear of breaking it

Ideas for improving the course and making it more useful	More hours Easy instructions Support and guidance Repetition and practice

## Appendix

As detailed below, we show 25 simple questions about your opinion on computers.

Please answer honestly and by yourself.

For each question, choose the option that fits your way of thinking.

A lot: 4
Quite a lot: 3
Not much: 2
Not at all: 1
(1) Using computers makes me nervous.
(2) Using computers can be fun.
(3) I like using the computer.
(4) Using the computer can be boring.
(5) Using the computer makes me feel clumsy.
(6) I'm afraid of using the computer.
(7) I think using the computer can be pleasant.
(8) Using the computer makes me feel valuable.
(9) I think learning to use the computer is something worthy.
(10) I think computers bring people together.
(11) I think using computers is very difficult.
(12) I think computers are too important in the world.
(13) I think computers are too complex.
(14) I think computers make your life easier.
(15) I think I have difficulties understanding computers.
(16) I'm worried about messing up the computer.
(17) I think computers grow people apart.
(18) I think computers make your life more complicated.
(19) I trust myself using a computer.
(20) I think using the computer takes a lot of dedication.
(21) I think it is hard to control what the computer does.
(22) Computers make me feel like I'm not up to date.
(23) It's easier to learn new things by using the computers.
(24) Using the computer allows you to interact with family and friends.
(25) Using the computer helps dealing with paperwork.
